# Flow Behaviour of Liquid and Gaseous Dielectrics and Debris Transport in the Inter-Electrode Gap of Micro-EDM Milling: A CFD Study

**DOI:** 10.3390/mi17060747

**Published:** 2026-06-20

**Authors:** Mohammad Bigdeli, Francesco Giovanni Modica, Valeria Marrocco, Irene Fassi

**Affiliations:** 1 Institute of Intelligent Industrial Technologies and Systems for Advanced Manufacturing, National Research Council of Italy-STIIMA-CNR, Via P. Lembo 38/F, 70124 Bari, Italy; mohammad.bigdeli@stiima.cnr.it (M.B.); francesco.modica@stiima.cnr.it (F.G.M.); 2 Institute of Intelligent Industrial Technologies and Systems for Advanced Manufacturing, National Research Council-STIIMA-CNR, Via A. Corti 12, 20133 Milan, Italy; irene.fassi@stiima.cnr.it

**Keywords:** micro-EDM milling, dielectric fluid, inter-electrode gap (IEG), k–ω turbulence model, debris transport, particle tracing, flushing efficiency

## Abstract

This study presents a transient computational fluid dynamics (CFD) analysis of dielectric flow behaviour and debris transport in micro-EDM milling, considering the effects of dielectric properties, inter-electrode gap (IEG) size (20–30 µm), and tool rotational speed (400–850 rpm). Four dielectric media, nitrogen gas, deionized water, HEDMA111 EDM oil, and sunflower seed oil, were investigated using a two-dimensional FEM-based model coupled with particle tracking simulations to evaluate debris mobility within the machining region. The results demonstrate that dielectric properties, particularly viscosity, strongly influence hydrodynamic behaviour and particle transport within the IEG. Under the adopted equal mass flow rate condition, nitrogen gas exhibited the highest flow velocities and the fastest debris evacuation due to the combined effects of its low viscosity and the resulting higher inlet velocity. Deionized water and HEDMA111 oil exhibit comparable intermediate behaviour, indicating that moderate viscosity variations within liquid dielectrics do not significantly alter the overall flow regime. In contrast, sunflower seed oil generates the most damped flow conditions, with reduced velocities and prolonged particle residence due to increased viscous resistance. Variations in IEG size produce only minor changes in evacuation efficiency compared with the dominant influence of dielectric properties, while tool rotational speed primarily affects velocity magnitude without altering qualitative transport behaviour.

## 1. Introduction

Micro-electrical discharge machining (µ-EDM) is a non-contact technology whose thermoelectric material removal mechanism, induced by electric discharges, enables the manufacturing of hard and brittle materials that are difficult to process using conventional cutting methods [1,2]. Among micro-EDM approaches, micro-EDM milling plays a primary role in machining 2D and 3D freeform features with the high precision and accuracy required for applications such as aerospace (e.g., to develop mm-wave and sub-THz antenna systems for future links in the D-band, G-band, and above), biomedical devices, and microfluidic systems.

Especially in micro-EDM milling, tool path and tool wear must be carefully controlled to guarantee machining stability, process performance, and final feature accuracy. To this end, dielectric flow plays a critical role in the inter-electrode gap (IEG). Indeed, since its size typically spans from a few micrometres (µm) to 25–50 µm, this extremely small space may potentially create process instability due to secondary discharges, arcing, and shorts. These phenomena are induced by inefficient dielectric flushing of debris [3], resulting in a reduced material removal rate (MRR), increased tool wear ratio (TWR), and increased surface roughness of the machined micro-features [4,5,6,7,8,9]. In this regard, Karthikeyan et al. [9] reported that micro-EDM milling of channels revealed that tool rotation and flushing, which influence centrifugal and viscous forces, induce modifications in the flow of molten metal. As a result, non-uniform resolified layers with uneven thicknesses were found on the sides of the micro-channels, causing edge tapering. Therefore, during micro-EDM milling, the dielectric fluid not only electrically insulates the electrodes between discharges during plasma breakdown and removes heat from the discharge zone, but also significantly contributes to process stability by transporting debris away from the active machining region in conjunction with the tool motion.

Generally, traditional hydrocarbon-based dielectrics, such as kerosene or HEDMA-type fluids, remain widely used due to their good insulation and flushing capabilities, which also promote increased MRR, although at the expense of tool wear. However, alternative dielectrics are increasingly being explored to address environmental and safety concerns while ensuring good micro-EDM performance [10,11]. For instance, deionized water has garnered attention for fine-feature machining, as it improves environmental compatibility, reduces TWR during short-pulse machining regimes [12] compared to hydrocarbon oils [13,14], and enhances material removal when a high open voltage and capacitance are used [15]. More recently, bio-based oils, such as vegetable oils [16,17], have been proposed as sustainable alternatives; however, their higher viscosity may significantly alter flow behaviour and debris transport within narrow gaps. Liquid–gas mixtures or powder-mixed dielectrics [18] have been shown to be effective in improving MRR, although this effect was only observed in roughing regimes.

In addition to liquid dielectrics, gaseous and gas-assisted dielectric media have also been investigated as alternative approaches to improve debris evacuation and process sustainability in EDM-based machining. In dry EDM, a gaseous dielectric replaces conventional liquid media, while near-dry EDM employs a gas–liquid mixture to enhance flushing efficiency and cooling performance. These approaches reduce the environmental impact associated with hydrocarbon oils and can significantly improve debris removal from the inter-electrode gap through high-velocity gas flow [19]. Experimental investigations have demonstrated that gas-based dielectric systems can achieve higher material removal rates and reduced tool wear under appropriate flushing and electrode motion conditions, particularly when pressurised gas or gas–liquid mixtures are introduced through tubular electrodes [17,20]. Furthermore, the lower viscosity and density of gaseous media promote rapid momentum transfer and efficient particle transport within confined gaps, potentially altering hydrodynamic behaviour compared with conventional liquid dielectrics.

The dielectric flow and debris effects become significant in micro-EDM milling, where strict lateral confinement and tool rotation generate highly complex flows and pressure fields within the small IEG. However, characterising such phenomena directly in the IEG remains challenging, as it requires complex equipment and post-processing. For instance, Shah et al. [21] presented an in-house setup that utilised high-speed photography and image processing techniques to monitor and analyse debris dynamics in real-time during micro-EDM dressing. Specifically, to understand the physics underlying debris exclusions under dielectric motion, the authors employed a COMSOL Multiphysics 6.0 model to simulate debris flow behaviour in the discharge gap. From this perspective, modelling approaches based on computational fluid dynamics (CFD) have been demonstrated to play a crucial role in predicting and capturing such phenomena at the microscale.

In this regard, a preliminary investigation by our group [22] examined dielectric flow and debris trajectories in micro-EDM milling using a 2D CFD model with laminar flow approximation: in particular, the findings highlighted the strong effect of rotational tool speeds on HEDMA111 oil flow uniformity and debris migration with different IEG sizes, from 20 to 30 µm. Mullya et al. [23,24,25] proposed 2D CFD models based on the finite element method (FEM) and coupled with Discrete Particle Tracking (DPT) to analyse the effects of tool rotation, cylindrical and slotted tools on kerosene flow behaviour and debris motion along the IEG of micro-EDM milling. The analyses accounted for variable gap sizes (30 to 50 µm), inlet nozzle velocity, and tool diameter. The numerical results showed that reducing the IEG size and increasing the rotational tool speed generally enhances the local dielectric velocity near the rotating tool surface. Additionally, the authors observed that recirculation zones and eddy structures that form behind the cylindrical tool act as secondary stirrers, influencing the redeposition and ejection of debris within the microchannel. Closely related studies [26,27] by the same authors used a similar 2D CFD framework coupled with a Discrete Phase Model (DPM) to investigate debris accretion in low-viscosity EDM oil for a fixed 50 µm IEG, considering variable particle size, injection position, tool rotational speed, and shapes. The main findings were that at low speeds particles underwent multiple recirculation loops before depositing on the workpiece, whereas higher speeds promoted entrainment within the vortex behind the tool and improved flushing. Furthermore, accretion patterns are sensitive to injection location, particle size, and flow conditions. In contrast, well-designed slots on tools have demonstrated the ability to enhance local velocity, reduce debris accumulation, and improve material removal rates by up to 150%. In this regard, an additional CFD analysis [28] reported that the slot geometry, jet velocity, rotation speed, and gap size strongly influence flushing behaviour and particle transport, with debris trajectories being highly sensitive to injection location and flow-induced cooling. Across their studies [23,24,25,26,27,28], Mullya et al. employed a low-Reynolds number RANS turbulence model for kerosene dielectric flow and reported that tool rotation within ultra-confined channels (30–50 μm) generates local flow disturbances, recirculation structures, and micro-scale eddies along the tool trajectory. The turbulence formulation also enabled the incorporation of turbulent dispersion effects into the DPM, providing an enhanced description of debris transport in the confined inter-electrode region.

Ge et al. [29] employed a CFD model coupled with DPM to analyse the dielectric kerosene flow behaviour and debris motion during micro-EDM drilling of a blind hole in Cf-ZrB2-SiC ceramic under disturbed flow conditions induced by electrode-assisted (EA) oscillation. Their results demonstrated that introducing controlled electrode motion significantly increased the dielectric velocity, suppressed low-velocity regions, and promoted vortex formation within the machining gap, thereby enhancing debris mobility and reducing the likelihood of secondary discharges.

Despite the utility of CFD models in providing insights into micro-EDM flushing behaviour, the literature focus has been limited to the evaluation of qualitative flow and redeposition trends under specific conditions, such as fixed gap sizes (30–50 μm) and a single dielectric fluid, thereby neglecting quantitative debris retention metrics and a comparative assessment across different dielectric properties.

A step forward was proposed by Guo et al. [30] and Wang et al. [31], who considered the flow dynamics and debris movement in different dielectric fluids, although in the context of micro-EDM drilling of micro-holes. In particular, Guo et al. [30] investigated debris transport during EDM of high-aspect-ratio small holes, considering different dielectric fluids, including kerosene, deionized water, and sunflower seed oil. The authors observed that the escape rate of corrosion residues from the discharge gap worsens as machining time, current, and machine depth are increased. Furthermore, dielectric properties, particularly viscosity and flow resistance, strongly influence debris lifting and evacuation efficiency. In particular, sunflower seed oil and water exhibited higher escape rates than kerosene, confirming that vegetable oils can serve as a sustainable alternative to kerosene. Wang et al. [31] examined the micro-EDM performance for machining holes as small as 1 mm in diameter, using vegetable oils. To this end, a CFD model coupled with a DPM showed that low-viscosity plant oils significantly increased particle escape rates and improved debris stability within the machining gap. Furthermore, experimental validations confirmed that MRR increased with lower-viscosity dielectrics, as was noticeable for sunflower seeds (MRR = 0.8552 mm³/min), rapeseed (MRR = 0.4603 mm³/min), and soybean oils (MRR = 0.3718 mm³/min).

The literature indicates that the fundamental flow characteristics and debris-transport mechanisms have been investigated mainly in relation to liquids. However, few works have focused on the use of gaseous dielectrics or on their fluid dynamics behaviour in micro-EDM milling operations. In this regard, it is worth noting that different dielectrics exhibit distinct thermophysical properties, particularly viscosity and density, which influence the local flow field and debris transport, particularly within smaller IEGs.

Therefore, the present work aims to systematically assess how differences in dielectric properties, particularly viscosity and density, affect local flow behaviour and debris transport under identical micro-EDM milling conditions. The analyses were performed using 2D CFD simulations incorporating a turbulence model and coupled with multiphase particle tracing (MPT) for variable rotational tool speeds (400, 600, and 850 rpm) and inter-electrode gaps (20, 25, and 30 µm). The standard k–ω formulation was chosen to provide robust near-wall treatment and improved representation of local shear layers and strong velocity gradients generated by tool rotation within the confined inter-electrode gap while maintaining numerical robustness across the broad range of dielectric viscosities and densities investigated in this work. Specifically, four dielectric media were considered in the simulations: nitrogen gas, deionized water, HEDMA111 oil, and sunflower seed oil. Beyond analysing velocity fields, the MPT framework was employed to quantitatively evaluate debris transport and retention through particle half-life and retained fraction as functions of dielectric type, IEG size, and rotational speed.

## 2. CFD Analysis: 2D FEM-Based Model and Governing Equations

### 2.1. Computational Geometry and Boundary Conditions

**Figure 1:**
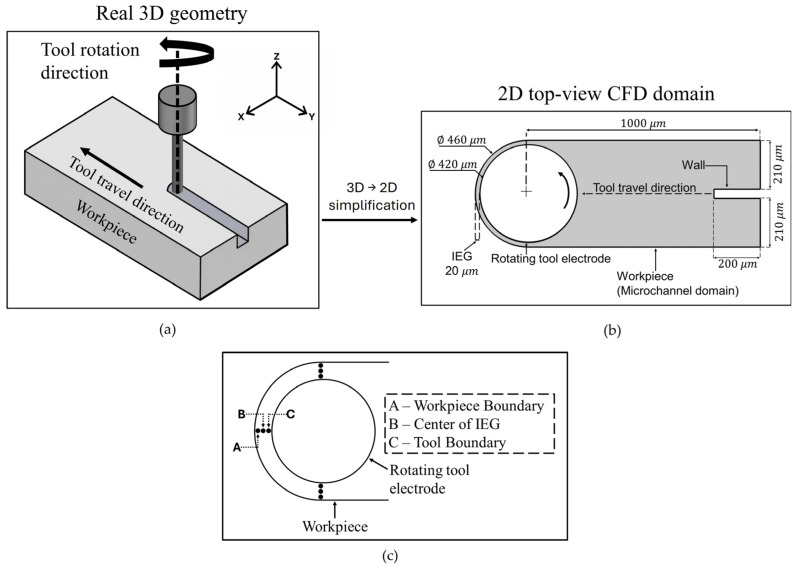
Representation of the modelling framework: (**a**) actual three-dimensional micro-EDM milling configuration; (**b**) simplified two-dimensional top-view computational domain adopted for the CFD simulations following the 3D to 2D simplification procedure showing the microchannel geometry and a representative inter-electrode gap (IEG) of 20 µm; (**c**) sampling locations used for flow analysis within the IEG. Points A, B, and C denote the workpiece boundary, gap centre, and tool boundary, respectively.

A transient FEM-based two-dimensional (2D) analysis was developed in COMSOL Multiphysics (Figure 1) to model the dielectric fluid flow during µ-EDM milling of a microchannel with a length of 1000 µm. Figure 1a illustrates the actual three-dimensional micro-EDM milling configuration, while Figure 1b presents the corresponding simplified two-dimensional top-view computational domain adopted in the present study to investigate the dominant lateral flow and debris-transport behaviour within the inter-electrode region. To analyse the spatial variation of the flow characteristics within the IEG, the gap was divided into a set of uniformly distributed sampling locations, as shown in Figure 1c. Points A, B, and C correspond to the workpiece boundary, mid-gap location, and tool boundary, respectively, and were used for detailed velocity evaluation.

Although dielectric flow within the IEG is inherently three-dimensional due to tool rotation and the associated complex flow structures, the present study adopts a two-dimensional representation to provide a computationally efficient framework for evaluating the influence of dielectric properties, IEG size, and rotational speed on flow and debris transport, aligned with other studies already reported in the literature [22,23,24,25,26,27,28]. The selected 2D top-view configuration captures the dominant lateral transport mechanisms and shear-driven effects and remains suitable for analysing hydrodynamic and debris-transport trends within the investigated conditions.

The cylindrical tool electrode, with a diameter of 420 µm, rotates counterclockwise while maintaining a constant 20 µm IEG from the workpiece wall. For numerical implementation, the inlet and outlet boundaries are placed on opposite sides of a 200 µm separating wall. This wall serves only to stabilise the boundary condition definitions in the CFD model and does not represent any physical obstruction in the real machining process, nor does it alter the flow behaviour in the vicinity of the rotating tool. To facilitate comparison across different IEG configurations (20, 25, and 30 µm), larger gap sizes were generated by proportionally reducing the tool diameter while keeping the workpiece boundary fixed. Tool rotation was modelled through a prescribed tangential velocity applied at the tool boundary, enabling investigation of lateral flow characteristics without including tool feed motion or axial flushing.

**Figure 2:**
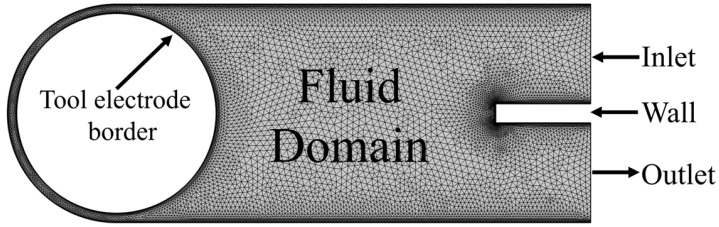
Mesh distributions generated using the physics-controlled mesh strategy implemented in COMSOL Multiphysics.

The computational domain was discretised using an unstructured mesh composed of triangular and quadrilateral elements (Figure 2, normal mesh configuration). A physics-controlled mesh strategy implemented in COMSOL Multiphysics was employed, resulting in local mesh refinement along the rotating tool boundary and the workpiece wall to accurately resolve the steep velocity gradients within the narrow IEG. As shown in Figure 2, the mesh distribution exhibits a higher element density in geometrically constrained regions and areas expected to experience strong flow variations, particularly near the rotating tool surface, to improve the resolution of local shear-driven flow structures and debris-transport behaviour. The normal mesh configuration was selected for all simulations because it provided an appropriate balance between numerical accuracy and computational efficiency. In this regard, the simulation’s sensitivity study to spatial discretisation is discussed in Section 3.2.1.

### 2.2. Governing Equations and Flow Model

To account for possible transitional and shear-driven flow effects within the narrow inter-electrode gap and provide robust near-wall treatment, improved flow resolution, and numerical robustness across the broad range of dielectric viscosities and densities investigated in this work, the standard k–ω model was adopted to provide closure for the Reynolds-averaged Navier–Stokes (RANS) equations. Thermal effects and gravitational forces are neglected due to the IEG’s small characteristic dimensions and the dominance of shear-driven flow in this configuration. The transient behaviour of the dielectric flow is governed by the incompressible RANS equations, which express the conservation of mass and momentum as

(1) ∇.u=0,

(2) $$\rho \left(\frac{\partial u}{\partial t}+u\;.\;\;\nabla u\right)=-\nabla p+\mu \nabla^{2}u,$$

where u = (u,v) is the velocity field in the 2D Cartesian plane; p is the pressure; and ρ and *µ* denote the density and dynamic viscosity of the dielectric fluid, respectively [32,33]. For completeness, the momentum equations in component form are

(3) $$\rho \;\left(\frac{\partial u}{\partial t}+u\frac{\partial u}{\partial x}+v\frac{\partial u}{\partial y}\right)=-\frac{\partial p}{\partial x}+\mu \left(\frac{\partial^{2}u}{\partial x^{2}}+\frac{\partial^{2}u}{\partial y^{2}}\right)$$

(4) $$\rho \;\left(\frac{\partial v}{\partial t}+u\frac{\partial v}{\partial x}+v\frac{\partial v}{\partial y}\right)=-\frac{\partial p}{\partial y}+\mu \left(\frac{\partial^{2}v}{\partial x^{2}}+\frac{\partial^{2}v}{\partial y^{2}}\right).$$

The flow regime was characterised using the Reynolds number, defined as

(5) $$Re=\;\frac{\rho \;U\;L}{\mu },$$

where ρ and μ are the density and dynamic viscosity of the dielectric fluid, respectively, while U is a characteristic velocity and L is a characteristic length scale of the flow.

To provide a representative estimate of the flow regime within the IEG, the characteristic length scale, *L*, was set equal to the IEG size, as the dominant velocity gradients, wall-shear effects, and particle transport mechanisms occur within this characteristic region, which controls the flow dynamics and debris evacuation process. The characteristic velocity was estimated as the sum of the inlet velocity and the tangential velocity induced by tool rotation:

(6) $$U=\;U_{inlet}+U_{tool},$$

where the tangential tool velocity is given by

(7) $$U_{tool}=\omega R=\frac{2\pi N}{60}R,$$

where N denotes the rotational speed (rpm), R the tool radius, and ω the corresponding angular velocity of the rotating tool. For the representative high-shear operating condition of 850 rpm and a tool radius of 210 µm, the tangential tool velocity was approximately 0.0187 m/s. Using the most demanding operating condition investigated in this study (IEG = 20 µm), representative Reynolds numbers of approximately 0.39, 0.16, 0.05, and 1.12 were obtained for deionized water, HEDMA111 oil, sunflower seed oil, and nitrogen gas, respectively. These values indicate that the investigated flow remains in the low-Reynolds-number regime despite the substantial differences in dielectric density and viscosity. Additionally, as reported by Mullya et al. [23,24,25,26,27,28], studies employing low-Reynolds-number RANS turbulence formulations for micro-EDM milling demonstrated that tool rotation generates local shear layers and strong velocity gradients within confined inter-electrode regions. These studies further showed that rotating tool motion significantly influences dielectric flow behaviour and debris transport within narrow microchannels. Therefore, the standard k–ω formulation was adopted in the present work to provide robust near-wall treatment and numerical stability across the broad range of dielectric viscosities and densities investigated. Furthermore, simplified assumptions regarding the thermophysical properties of dielectrics, such as constant fluid properties (density and dynamic viscosity), negligible temperature effects due to the controlled temperature range during machining typical of conditioned dielectric fluid, and small pressure variations within the IEG, were applied to the model.

### 2.3. Numerical Parameters and Dielectric Properties

The principal numerical settings and simulation parameters adopted in the present CFD flow simulations and particle transport analyses are summarised in Table 1, including fluid properties (Table 2), tool kinematics, inlet conditions, particle parameters, solver settings, and the time ranges used for both the flow and particle tracking simulations. Four dielectric media were considered in this study: nitrogen gas, deionized water, sunflower seed oil, and HEDMA111 oil. Their dynamic viscosity and density were used as input parameters in the CFD simulations to model the hydrodynamic behaviour within the inter-electrode gap (IEG) [28,34]. For the liquid dielectrics (deionized water, sunflower seed oil, and HEDMA111 oil), a constant inlet velocity of 0.1 cm/s was imposed at the inlet boundary. In contrast, since the gaseous dielectric case had a density nearly three orders of magnitude lower than the liquid cases, nitrogen gas at 20 °C was introduced at an equivalent mass flow rate to the deionized water reference case. Since the inlet cross-sectional area remained unchanged, the corresponding gas inlet velocity was calculated from the mass flow rate relation $\dot{m}=\rho uA$, ensuring that the same mass flow rate was supplied at the inlet for both liquid and gaseous cases.

### 2.4. Model Limitations and Assumptions

The present modelling framework involves several assumptions and limitations, which are summarised below:

- The model is two-dimensional. Consequently, certain three-dimensional flow features, such as out-of-plane flow structures and secondary recirculation effects, are not represented, which may affect the detailed local flow characteristics.
- Thermal effects, bubble dynamics, evaporation, and discharge-plasma physics were not included. These phenomena may significantly influence dielectric behaviour and local debris transport under practical machining conditions due to discharge-induced heating and transient multiphase interactions.
- Fluid properties were assumed to be constant and temperature-independent. As a result, local variations in density and viscosity caused by temperature changes during machining were not considered, potentially influencing the predicted flow behaviour under actual processing conditions.
- Particle interactions such as collisions, adhesion, and agglomeration were neglected. These interactions may alter particle trajectories and retention behaviour, particularly at higher local debris concentrations.
- Particle transport metrics are based on domain-level tracking and do not represent the full complexity of real machine flushing paths. Therefore, the predicted particle retention metrics should be interpreted primarily as comparative indicators rather than exact representations of practical debris evacuation behaviour.

**Figure 3:**
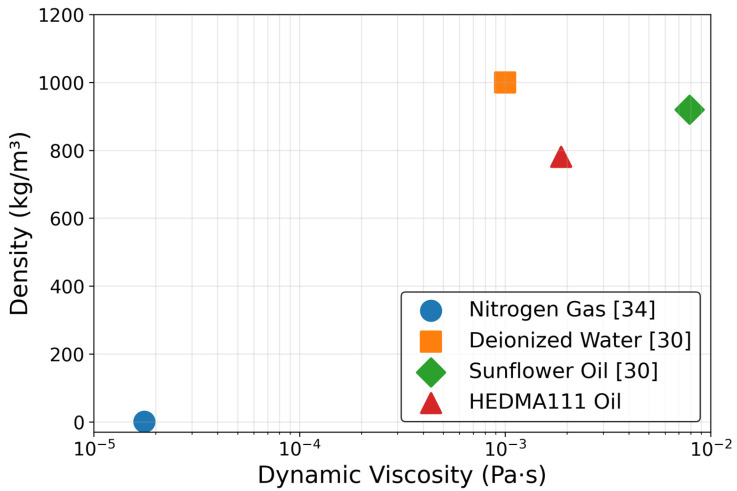
Dynamic viscosity (logarithmic-scaled) vs. the density of the dielectric fluids considered in this study, compiled from Refs. [30,34].

The considered dielectric media span a broad range of viscosities and densities, from extremely low-density gaseous conditions (nitrogen gas) to higher-viscosity liquid dielectrics, as shown in Figure 3 and Table 2. In particular, nitrogen gas exhibits a substantially lower dynamic viscosity and density nearly three orders of magnitude lower than that of the investigated liquid dielectrics. Given nitrogen gas’s extremely low viscosity and density, simulations were performed by setting a mass flow rate equal to that of the liquids to match the inertial effects observed in the liquid flow. This assumption enables a physically consistent and systematic comparison of dielectric media with significantly different densities and viscosities and allows the assessment of fluid properties’ influence on hydrodynamic behaviour and debris transport within the IEG.

## 3. Results and Discussion

### 3.1. Velocity Field Characteristics in the IEG

The dielectric flow behaviour within the IEG is analysed under identical µEDM milling operating conditions, with particular emphasis on the transient evolution of the velocity field and the corresponding quasi-steady characteristics. The analysis considers variations in IEG size and tool rotational speed (Table 1), as well as the four dielectric media whose thermophysical properties are summarised in Table 2.

**Figure 4:**
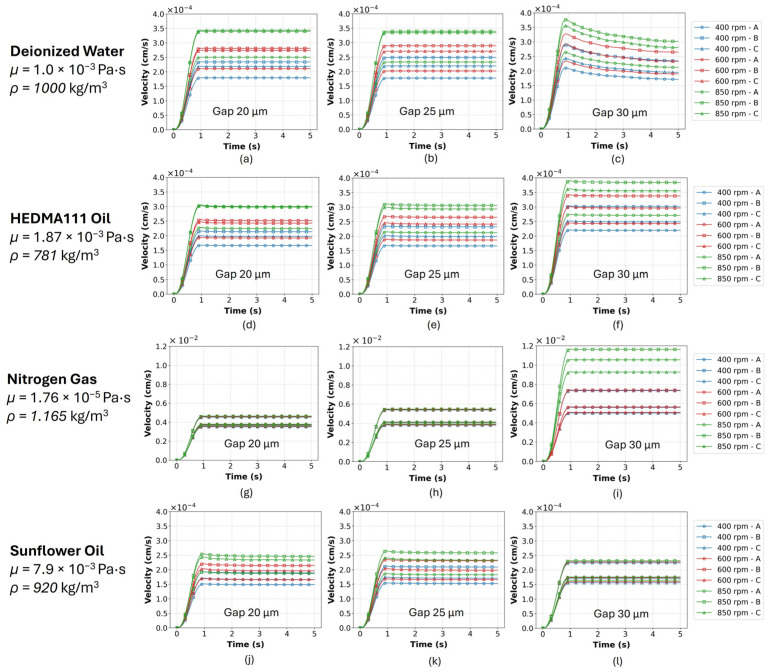
Velocity evolution at different rotational speeds and different control points (A, B, and C) for: deionized water (inter-electrode gap sizes of (**a**) 20 µm, (**b**) 25 µm, and (**c**) 30 µm), HEDMA111 oil (inter-electrode gap sizes of (**d**) 20 µm, (**e**) 25 µm, and (**f**) 30 µm), nitrogen gas (inter-electrode gap sizes of (**g**) 20 µm, (**h**) 25 µm, and (**i**) 30 µm) and sunflower seed oil (inter-electrode gap sizes of (**j**) 20 µm, (**k**) 25 µm, and (**l**) 30 µm).

Figure 4a–c illustrate the evolution of velocity at points A, B, and C for deionized water at varying IEG sizes and tool rotational speeds; as can be inferred from the curves, the fluid exhibits rapid acceleration and a short stabilisation time, about 1 s. The highest quasi-steady velocity levels, reaching ≈ $3.5\times 10^{-4}\;$cm/s, occur at point B for all investigated gap sizes and are consistently observed at 850 rpm. This behaviour reflects the location of point B within the main shear-driven flow region, where momentum transfer from the rotating tool is most effective. For the largest IEG (30 µm), the fluid behaviour change, showing a mild velocity overshoot followed by a gradual decay, can be observed before the flow reaches a quasi-steady regime.

Figure 4d–f show that, similarly to deionized water, HEDMA111 oil also develops smooth and stable velocity profiles across all IEG sizes, reaching a quasi-steady velocity state after about 1 s; it also displays mean velocities between approximately $1.7\times 10^{-4}$ and $3.8\times 10^{-4}\;cm/s$, whereas the highest velocities are recorded for a rotation speed of 850 rpm at point B for all investigated gap sizes. While for the 20 µm and 25 µm gaps the fluid shows a similar range of velocities, in the case of the largest IEG (30 µm) the fluid velocities are slightly amplified. Probably, thanks to the higher viscosity of HEDMA111 oil compared to deionized water, the mild velocity overshoot disappears.

Due to the initial condition, the nitrogen gas results in Figure 4g–i show considerably higher velocity levels than those observed for the liquid dielectric media, reaching approximately $\left(0.4–1.2\right)\times 10^{-2}\;cm/s$ depending on the IEG size and rotational speed, with the maximum values at 850 rpm. The velocity profiles accelerate rapidly and stabilise within about 1 s at all monitoring points (A–C), indicating fast establishment of the shear-driven flow and efficient momentum transfer from the rotating tool. Increasing the IEG from 20 to 30 µm increases the velocity, due to the combined effects of nitrogen’s low viscosity; the substantially higher inlet velocity associated with the equal mass flow rate condition; and reduced confinement, which promotes stronger mixing and particle mobility within the gap.

Figure 4j–l present the evolution of velocity for the highly viscous sunflower seed oil. Compared with the other dielectric fluids, the velocity magnitudes remain relatively low, in the approximate range of $\left(∼1.5–2.6\right)\times 10^{-4}\;cm/s$, and show limited sensitivity to both rotational speed and IEG size. The profiles increase smoothly and stabilise rapidly without noticeable transient peaks, reflecting the strong viscous damping associated with the high dynamic viscosity. This behaviour indicates stable but weakly accelerated flow within the IEG.

Considering the time required to reach steady-state flow, the viscous oils exhibit smooth, largely monotonic velocity evolution and rapid stabilisation. However, their dependence on IEG size differs: HEDMA111 oil shows increased quasi-steady velocities at the largest gap (30 µm), whereas sunflower seed oil exhibits a slight reduction in velocity at 30 µm compared with the 20–25 µm cases, indicating weaker momentum transfer under highly viscous conditions. In contrast, deionized water, particularly at 30 µm, exhibits a more pronounced transient response, with a mild overshoot followed by gradual decay before reaching quasi-steady conditions. Nitrogen gas stabilises rapidly and demonstrates the strongest sensitivity to gap enlargement, exhibiting substantially higher velocity levels at 30 µm under the adopted equal mass flow rate condition.

**Figure 5:**
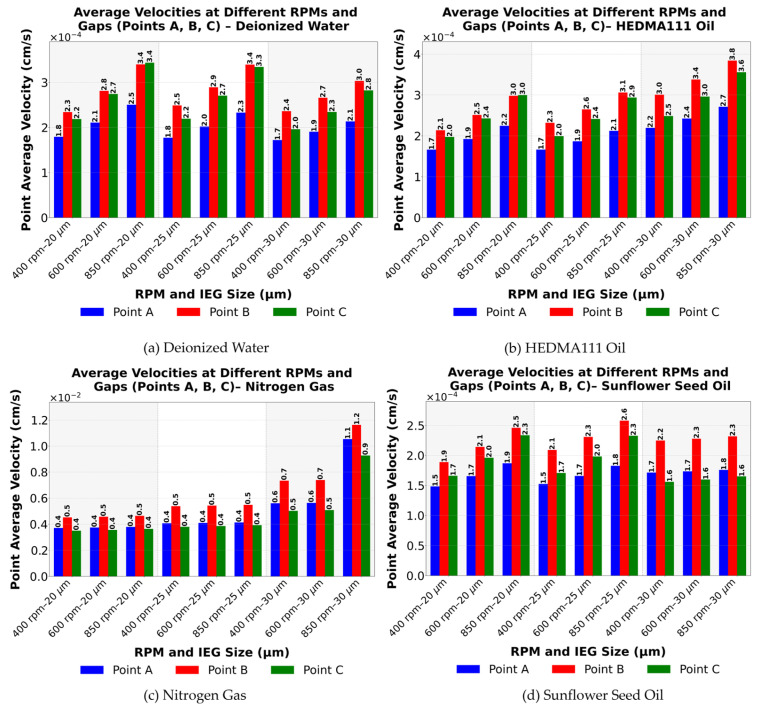
Steady and quasi-steady average velocities across RPMs and IEGs for (**a**) Dieonized water, (**b**) HEDMA111 Oil, (**c**) Nitrogen Gas, and (**d**) Suflower Seed Oil.

Figure 5 compares the steady or quasi-steady (late-time) average flow velocities within the IEG at rotational speeds of 400–850 rpm and gap sizes of 20, 25, and 30 µm for the four investigated dielectric media at the control points (A, B, and C). The reported velocities are obtained by averaging over the final 20% of the simulation time, after the initial transient decays. The corresponding quantitative ranges are summarised in Table 3.

Across all examined conditions, a consistent hierarchy in velocity magnitude can be observed: $V_{nitrogen}$ ≫ $V_{HEDMA111}\;\approx$ $V_{water}$ > $V_{sunflower}$, demonstrating the influence of dielectric properties under the adopted inlet-condition strategy. It should be emphasized that the high velocity magnitude observed for nitrogen gas is not solely attributable to its low viscosity. Under the adopted equal mass flow rate normalisation strategy, the very low nitrogen density required a substantially higher inlet velocity (83 cm/s) to maintain a mass flow rate comparable to that imposed in the liquid dielectric cases. Consequently, the elevated flow velocity of nitrogen arises from the combined effects of its fluid properties and the imposed inlet-condition normalisation.

Comparing the results for the liquids, two different behaviours can be identified: for the IEG strictly related to micro-EDM processing, from 20 µm to 25 µm, the velocity between the tool electrode and the workpiece is mainly unchanged; on the contrary, when IEG is equal to 30 µm, the flow behaviours change, displaying some overshoot related to dynamic viscosity and density.

For all fluids:

- Increasing the rotational speed from 400 to 850 rpm results in higher average velocities within the IEG; however, the sensitivity to rpm is strongly fluid-dependent. Nitrogen gas exhibits the most pronounced scaling with rotational speed, reflecting inertia-dominated dynamics in which rotational forcing is efficiently transmitted to the fluid due to its low viscosity and reduced flow resistance. Deionized water and HEDMA111 oil display a more moderate dependence on rpm, indicative of mixed viscous–inertial behaviour. In contrast, sunflower seed oil shows only a weak response to increasing rotational speed, as its high viscosity suppresses near-wall shear amplification and attenuates velocity growth.
- The influence of IEG size also varies with the dielectric medium. Increasing the IEG for nitrogen gas and HEDMA111 oil generally leads to higher steady or quasi-steady velocities, primarily due to reduced geometric confinement and the availability of a larger effective cross-section flow within the gap. By comparison, deionized water exhibits only a weak dependence on IEG size, with modest variations across the investigated gaps, suggesting a balance between geometric expansion and shear-driven acceleration. Sunflower seed oil shows relatively small changes across gap sizes, with a slight reduction at the largest IEG, indicating that viscous diffusion dominates over inertial transport and partially masks geometric sensitivity.

Higher average velocities within the IEG reflect stronger shear-driven fluid motion and an increased tendency for particles to move within the gap, whereas lower velocities correspond to more viscously damped flow conditions associated with reduced particle mobility and greater spatial stability.

The predicted hydrodynamic trends are consistent with observations reported in previous CFD investigations [23,24,25], which show that increasing tool rotational speed up to 800 rpm in the IEGs from 30 to 50 µm enhances dielectric flow velocity (ranging between 0.5 and 1.5 m/s) and promotes improved debris transport due to stronger shear-driven recirculation and secondary flow structures generated around the rotating tool. Conversely, the formation of micro-eddies at lower rpm values leads to higher debris retention. These studies also qualitatively agree with the present results, showing that local flow behaviour and debris migration are influenced by both gap size and inlet flow conditions, particularly under high-velocity flushing conditions. Furthermore, the strong sensitivity observed for the nitrogen gas case under equal mass flow rate normalisation is consistent with the fact that elevated inlet velocities can substantially intensify dielectric flow and improve debris-transport efficiency within the machining gap.

### 3.2. Particle Transport and Retention Analysis

**Figure 6:**
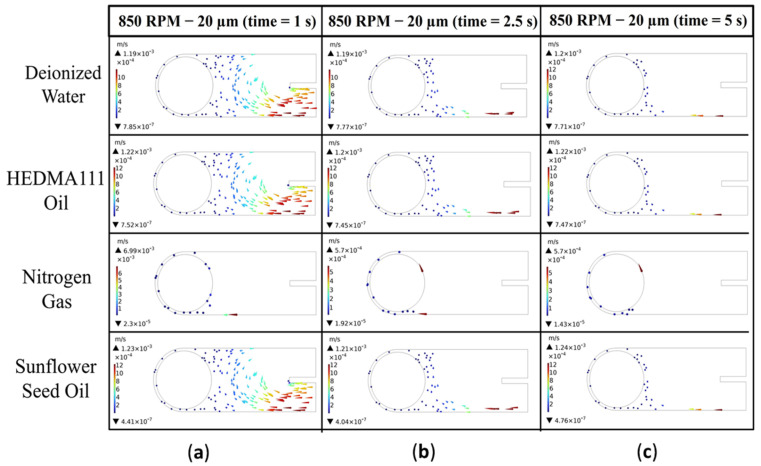
Particle trajectories for the four dielectric fluids at 850 rpm and a 20 µm inter-electrode gap (IEG) shown at (**a**) t=1 s, (**b**) t=2.5 s, and (**c**) t=5 s.

To describe the local mobility of debris within the IEG for each dielectric type, the particle trajectory snapshots at t =1 s, 2.5 s, and 5 s at 850 rpm for IEG = 20 µm are depicted in Figure 6.

Deionized water, HEDMA111 oil, and sunflower seed oil exhibit comparatively slow particle motion within the IEG. The broadly similar trajectories observed for these fluids indicate that moderate viscosity differences within the liquid range do not significantly alter the overall debris-transport behaviour.

In contrast, at t=1 s, nitrogen gas exhibits rapid particle transport within the IEG, with a larger proportion of particles already displaced toward the downstream outlet region than in liquid dielectrics. This behaviour continues at t=2.5 s and t=5 s, during which particles continue moving within the gap before leaving the computational domain, resulting in fewer particles remaining over time relative to the liquid media. The very low viscosity of nitrogen, combined with the higher inlet velocity associated with equal mass flow rate normalisation, enables efficient momentum transfer from the rotating tool, leading to higher particle mobility and faster evacuation. In liquid media, a larger fraction of particles remains in the downstream region at early times, and particle movement near the tool progresses more gradually than in nitrogen.

The observations in Figure 6 highlight a clear, fluid-dependent trade-off in debris-transport behaviour:

- Liquid dielectrics (deionized water, HEDMA111 oil, and sunflower seed oil) exhibit comparatively slower particle motion, with a greater proportion of debris remaining within the IEG and downstream micro-channel over time. Increasing viscosity further reduces particle mobility, particularly in sunflower seed oil.
- Nitrogen gas promotes rapid particle displacement within the IEG, with a large fraction of debris transported toward the downstream micro-channel at early times. Particles remain mobile as they traverse the gap before leaving the computational domain, resulting in a reduced residence time compared with the liquid dielectric media.

To quantitatively compare debris-evacuation efficiency across different dielectric fluids, IEG sizes, and rotational speeds, the time evolution of transported particles was analysed using the particle tracking method (PTM). For each simulation case, a fixed number of particles was randomly released into the computational domain at the initial simulation time and their trajectories were tracked throughout the transient simulation window (5 s). The number of particles still present inside the computational domain was evaluated at each recorded time step and they were classified as escaped once they crossed the outlet boundary of the micro-channel, while those remaining within the domain were considered retained.

The initial number of released particles is denoted as

(8) $$N_{0}=N(0)$$

where $N_{0}\;$represents the initial number of particles released into the computational domain at the beginning of the simulation. A key performance metric extracted from the particle tracking results is the particle half-life, defined as the time required for half of the particles to exit the machining zone:

(9) $$N\left(t_{1/2}\right)=\;\frac{N_{0}}{2}$$

where $t_{1/2}$ denotes the flushing half-time corresponding to 50% particle evacuation from the machining region. A shorter half-life indicates faster debris removal and, therefore, more effective flushing behaviour. In addition to the half-life, the percentage of particles that escaped in the computational domain at the final simulation time t=5 s was computed as

(10) $$\%\;Escaped\left(t\right)=\left(1-\frac{N(t)}{N_{0}}\right)\times 100.$$

where N(t) is the number of particles remaining inside the computational domain at time t and $N_{0}$ is the initial number of released particles. This framework provides a consistent quantitative basis for comparing debris-transport performance across all simulated operating conditions. For all simulation cases, an identical number of particles ($N_{0}=200$) was released randomly into the computational domain representing the inter-electrode region.

**Figure 7:**
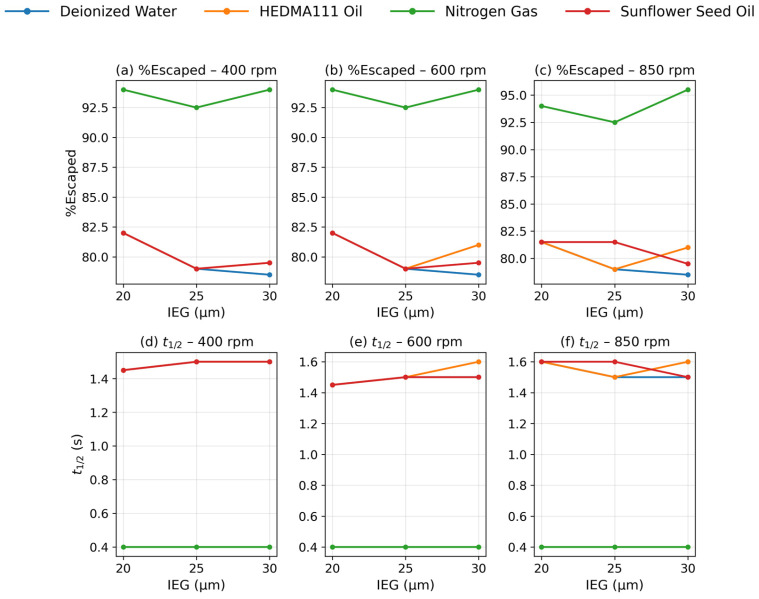
Summary of particle retention metrics for different dielectric fluids, IEGs, and tool rotational speeds: (**a**) escaped-particle percentage (% escaped) at 400 rpm; (**b**) escaped-particle percentage (% escaped) at 600 rpm; (**c**) escaped-particle percentage (% escaped) at 850 rpm; (**d**) flushing half-time ($t_{1/2}$) at 400 rpm; (**e**) flushing half-time ($t_{1/2}$) at 600 rpm; and (**f**) flushing half-time ($t_{1/2}$) at 850 rpm.

Figure 7 summarises the effects of the investigated process parameters, namely, dielectric fluid type, IEG size, and rotational speed, on the principal particle transport indicators extracted from the simulations, including the flushing half-time ($t_{1/2}$) and the corresponding percentages of escaped particles. These indicators provide a compact quantitative comparison of debris-transport performance across the investigated operating conditions, facilitating clearer differentiation among cases that may exhibit similar particle retention trends in the temporal curves.

**Figure 8:**
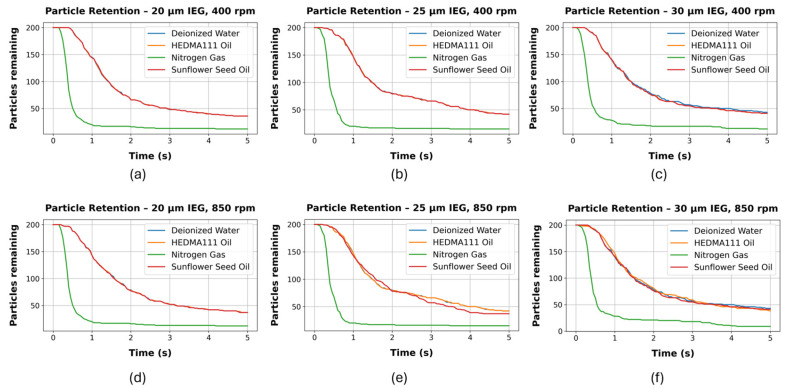
Particle retention curves for dielectric fluids at different IEGs and rotational speeds: (**a**) IEG = 20 µm and RPM = 400, (**b**) IEG = 25 µm and RPM = 400, (**c**) IEG = 30 µm and RPM = 400, (**d**) IEG = 20 µm and RPM = 850, (**e**) IEG = 25 µm and RPM = 850, and (**f**) IEG = 30 µm and RPM = 850.

Additionally, Figure 8 illustrates the temporal evolution of particle retention for the three investigated IEG values (20, 25, and 30 µm) and the rotational speeds (400 and 850 rpm). As shown, the influence of IEG size on the percentage of escaped particles varies depending on the dielectric medium. Nitrogen gas exhibits consistently high evacuation efficiency across all gap sizes, with a slight increase in the percentage of escaped particles when the IEG increases from 25 to 30 µm, indicating that efficient particle transport is promoted by the combined effects of low viscosity and the adopted equal mass flow rate inlet condition. HEDMA111 oil shows moderate sensitivity to gap size, with improved particle escape at 30 µm, particularly at higher rotational speeds (600 and 850 rpm), whereas sunflower seed oil presents only minor, non-monotonic variations due to its high viscosity. Deionized water exhibits nearly constant or slightly decreasing escaped percentages with increasing IEG, indicating weak dependence on geometric expansion within the investigated range.

These results reveal that the dielectric physical phase and viscosity exert a stronger influence on particle evacuation efficiency than moderate variations in IEG size. Considering the dielectric type, nitrogen gas achieves the fastest particle removal, characterised by the shortest flushing half-times (≈0.4 s) and the lowest residual particle counts across all operating conditions. In contrast, the liquids, deionized water, HEDMA111 oil, and sunflower seed oil, show comparatively slower removal, with longer half-times (≈1.4–1.6 s) and higher particle retention. Indeed, the differences among the liquid dielectrics remain moderate. These retention indicators quantify global particle removal rather than local debris mobility within the IEG, which is governed by the combined effects of dielectric properties, inlet conditions, and geometric confinement, followed by IEG size, whereas rotational speed plays a secondary role within the investigated range.

**Figure 9:**
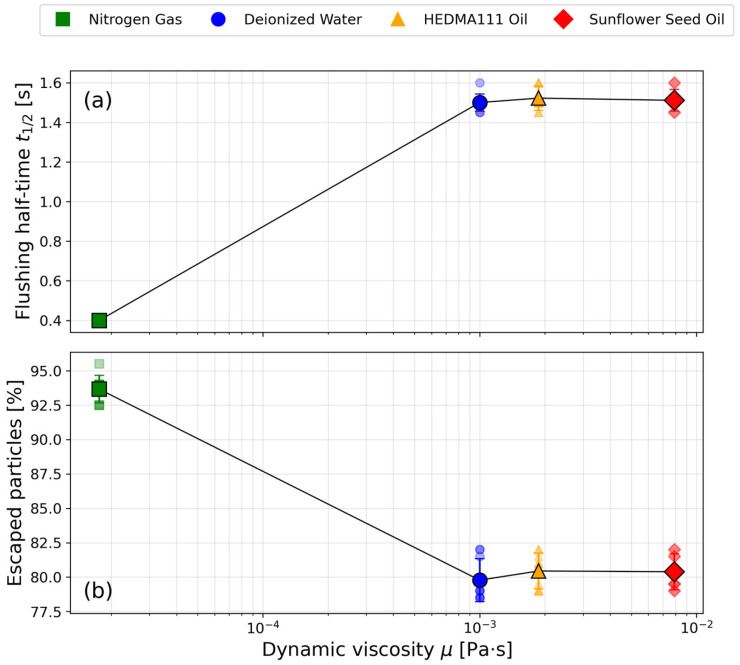
Influence of dielectric fluid dynamic viscosity (μ) on flushing performance: (**a**) flushing half-time ($t_{1/2}$); (**b**) % escaped particles. Larger markers denote means ± standard deviations for each dielectric fluid, whereas faint markers represent individual measurements obtained for different IEG sizes and tool rotational speeds.

The comparative plot in Figure 9 indicates that, under the investigated equivalent-mass flow conditions, the flushing behaviour of nitrogen gas is clearly separated from that of the liquid dielectrics. The gas phase yields the shortest flushing half-time and the highest escaped-particle fraction, confirming substantially enhanced flushing efficiency. In contrast, deionized water, HEDMA111 oil, and sunflower seed oil display relatively similar flushing metrics despite their differences in viscosity. This clustering of the liquid dielectric results suggests that viscosity alone does not fully govern debris-removal behaviour within the investigated operating range. Instead, the observed transport behaviour is likely influenced by the combined effects of viscosity, density, flow-field development, and operating parameters.

#### 3.2.1. Simulation Sensitivity to Mesh Generation, Temporal Discretisation, and Particle Size

To assess the results, additional simulations were performed to verify sensitivities to mesh size, temporal evolution, and particle diameters.

**Figure 10:**
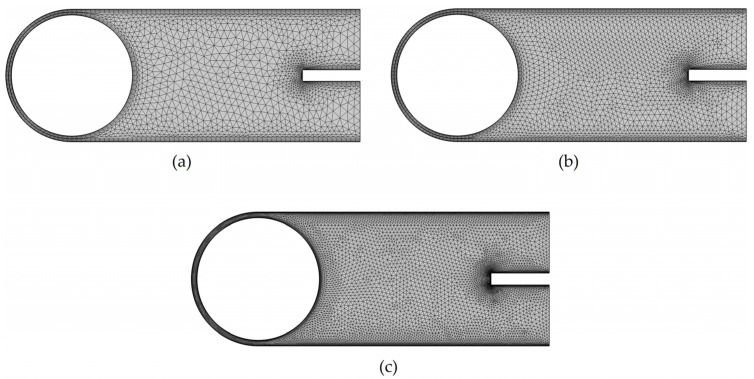
Mesh configurations used for the study: (**a**) coarse mesh, (**b**) normal mesh selected for subsequent simulations, and (**c**) fine mesh. The mesh distributions were generated using the physics-controlled mesh strategy implemented in COMSOL Multiphysics.

For spatial discretisation, a study involving three mesh densities, denoted as coarse, normal, and fine, was performed for the representative case of deionized water at a rotational speed of 850 rpm and an IEG of 20 µm. Figure 10 illustrates the corresponding mesh distributions within the computational domain, showing the variation in mesh density across the investigated discretisation levels, while Table 4 summarises the principal mesh characteristics.

The average element quality was evaluated using COMSOL’s element-quality metric, which assesses the geometric distortion of mesh elements relative to an ideal element shape. The quality value ranges from 0 to 1, where values closer to 1 indicate higher-quality elements with lower geometric distortion. As summarised in Table 4, the coarse mesh consisted of 4356 domain elements, the normal mesh contained 6678 elements, and the fine mesh included 16,778 elements. The average element quality remained approximately constant for all investigated meshes, with values close to 0.78–0.79, indicating acceptable mesh quality throughout the discretisation levels.

Considering the three mesh settings, the flushing half-time ($t_{1/2}$), the number of particles remaining within the computational domain at t=5 s, and the corresponding escaped-particle percentage were evaluated. These quantities were selected because they directly characterize debris-retention behaviour and flushing efficiency within the machining zone. The results, shown in Table 4, demonstrated progressive numerical convergence with mesh refinement. In particular, the difference between the normal and fine meshes was approximately 3.45% for the flushing half-time, whereas the remaining particle count and escaped-particle percentage at t=5 s showed negligible differences between the two meshes. This indicates that the principal particle transport predictions become weakly sensitive to additional mesh refinement beyond the normal discretisation level. Based on these results, the chosen normal mesh configuration (Figure 10b) was selected for all simulations, providing an appropriate trade-off between numerical accuracy and computational efficiency, while further refinement yielded only negligible changes in the principal particle transport indicators.

A temporal sensitivity analysis was also performed using the same representative case, and the corresponding results are summarised in Table 5. The temporal sensitivity assessment demonstrated that the principal particle retention indicators remained stable with temporal refinement. In particular, the predicted number of remaining particles and the escaped-particle percentage at t=5 s showed negligible differences between the normal and fine temporal discretisations, whereas the flushing half-time exhibited only moderate variation.

To evaluate the sensitivity of the particle tracking results, additional simulations were performed using the same representative case of deionized water at 850 rpm and an IEG of 20 µm and selecting particle diameters of 0.1, 1, 2, and 5 µm, aligned with debris sizes used numerically [25,26] and observed experimentally [32,33,35,36]. The obtained results showed negligible variations in both flushing half-time ($t_{1/2}\approx 1.4\;s$) and escaped-particle percentage at t=5 s (≈82%) across the investigated diameter range. These observations indicate that, under the present operating conditions, the predicted particle retention behaviour is weakly sensitive to particle diameter. This behaviour is attributed to the dominance of Stokes drag under the low-inertia microscale flow conditions, which causes the particles to closely follow the local fluid streamlines.

It should be noted that the present particle tracking framework represents an idealised debris-transport model in which the debris particles are treated as standardised spherical particles with drag behaviour based on the Stokes flow assumption. Consequently, the model is primarily intended for comparative trend analysis of flushing performance under different dielectric and operating conditions rather than exact prediction of real debris morphology and transport behaviour during practical micro-EDM machining.

#### 3.2.2. Effect of Inlet Normalisation Strategy on Nitrogen Gas Simulations

To further clarify the influence of the inlet-condition normalisation strategy on the nitrogen gas simulations, an additional comparison was conducted using the same inlet velocity as the liquid dielectric cases (0.1 cm/s). The obtained results were compared with the original nitrogen gas case based on equal mass flow rate normalisation and with the deionized water reference case.

**Figure 11:**
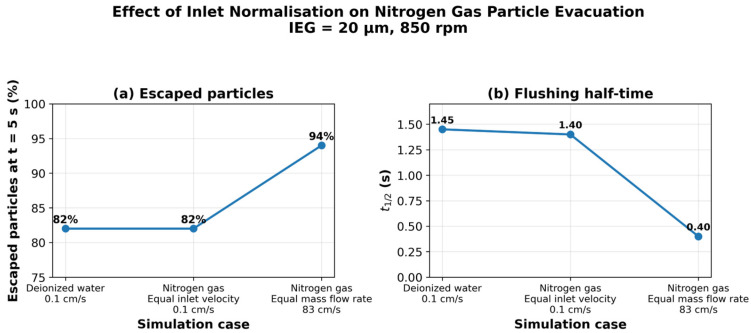
Effect of inlet-condition normalisation on nitrogen gas particle evacuation at an inter-electrode gap (IEG) of 20 µm and a rotational speed of 850 rpm: (**a**) escaped-particle percentage at t=5 s and (**b**) flushing half-time ($t_{1/2})$ for deionized water and nitrogen gas under equal inlet velocity and equal mass flow rate conditions.

Figure 11 further illustrates the influence of inlet-condition normalisation on the predicted nitrogen gas particle transport behaviour at an IEG of 20 µm and a rotational speed of 850 rpm. Under equal inlet velocity conditions (0.1 cm/s), nitrogen gas and deionized water exhibited nearly identical evacuation behaviour, with escaped-particle percentages of approximately 82% at t=5 s and flushing half-times ($t_{1/2})\;$of approximately 1.40–1.45 s. In contrast, the original nitrogen gas case based on equal mass flow rate normalisation produced substantially faster debris evacuation characterised by an escaped-particle percentage of approximately 94% and a significantly shorter flushing half-time of approximately 0.4 s. It must also be considered that, at the same inlet velocity, the fluid inertia effect is dramatically lower in the gaseous dielectric case due to the density being extremely lower than in the liquid cases, damping the low viscosity effect.

These results indicate that the enhanced evacuation performance observed in the original nitrogen simulations is strongly influenced by the substantially higher imposed inlet velocity required to maintain the same mass flow rate as the liquid dielectric reference case. Therefore, the gas–liquid comparison reflects the combined effects of dielectric properties and inlet-condition normalisation strategy rather than viscosity alone.

## 4. Conclusions

In this work, a transient 2D CFD model coupled with particle tracking analysis was developed to investigate the influence of dielectric properties, inter-electrode gap (IEG) size (20–30 µm), and tool rotational speeds (400–850 rpm) on flow behaviour in the IEG and debris transport in micro-EDM milling. To this end, four dielectric media, nitrogen gas, deionized water, HEDMA111 oil, and sunflower seed oil, were analysed to evaluate the combined effects of viscosity, density, and geometric confinement on the hydrodynamic conditions within the IEG. To account for possible transitional and shear-driven flow effects within the narrow IEG and provide robust near-wall treatment, improved flow resolution, and numerical robustness across the broad range of dielectric viscosities and densities investigated in this work, the simulations were run using the standard k–ω model based on Reynolds-averaged Navier–Stokes (RANS) equations. In this regard, simplified assumptions were also applied:

- Thermal effects and gravitational forces were neglected due to the IEG’s small sizes and the dominance of shear-driven flow in this configuration;
- Fluid properties (density and dynamic viscosity) are constant and temperature-independent;
- Temperature effects are negligible due to the controlled temperature range during machining;
- Pressure variations are small within the IEG.

Moreover, given nitrogen gas’s extremely low viscosity and density, the simulations were performed with a mass flow rate equal to that of the liquids.

Under these conditions, the results show that the dielectric phase (gas or liquid) and viscosity strongly influence both the intensity of the hydrodynamic field and particle transport behaviour. Lower-viscosity media generate higher velocities and faster particle acceleration, whereas higher-viscosity fluids increase hydraulic resistance and suppress flow motion. The principal findings can be summarised as follows:

- Nitrogen gas exhibits the highest flow intensity and the most efficient particle evacuation, but these effects are ascribed to the combined effects of its fluid properties and the higher inlet velocity (83 cm/s) resulting from the normalized inlet condition of the mass flow rate being equal to that set for the liquids. As a consequence, particle evacuation is higher (escaped-particle percentages of approximately 94%); the largest velocity magnitudes, the shortest flushing half-times (≈0.4 s), and the lowest residual particle counts across all operating conditions were also displayed. Conversely, under inlet velocity conditions equal to those of the liquids (0.1 cm/s), it was verified that nitrogen gas and deionized water exhibited nearly identical behaviour (escaped-particle percentages of approximately 82% and flushing half-times of approximately 1.40–1.45 s).
- Deionized water and HEDMA111 oil display similar hydrodynamic behaviour, with comparable velocity levels and particle removal characteristics, indicating that moderate differences in viscosity do not significantly alter the overall flow regime inside the IEG.
- Sunflower seed oil produces the most damped flow conditions, with lower velocity magnitudes and slower particle removal due to its high viscosity, which suppresses shear-driven motion and increases the residence time within the machining region.
- Variations in IEG size between 20 and 25 µm display a limited influence on particle evacuation efficiency, while the increase to 30 µm has a secondary influence, producing only modest changes in the percentage of escaped particles compared with the dominant effect of dielectric properties. Larger gaps modify local flow structures, but viscosity remains the primary parameter governing transport performance.
- Rotational speed increases velocity magnitude but does not significantly alter the qualitative debris-transport behaviour within the investigated range, indicating that fluid properties and geometric confinement play a more important role than tool speed under the present conditions.

It should be emphasised that the proposed model and results do not account for physical phenomena associated with the realistic micro-EDM process, including discharge-induced thermal effects, plasma dynamics, dielectric evaporation, bubble generation and collapse, local conductivity variations, particle–particle interactions, agglomeration, and adhesion effects. These phenomena may substantially influence local debris motion and dielectric behaviour, especially under actual micro-EDM milling conditions. Therefore, the present results should be interpreted primarily as a comparative assessment of hydrodynamic and particle transport behaviours of dielectric flushing performance within small IEGs and do not provide a fully comprehensive evaluation of dielectric suitability for the overall micro-EDM process.

Nonetheless, the present findings provide a physics-based understanding of how dielectric properties influence flushing mechanisms given the assumptions of the present CFD model and may support the interpretation of dielectric-flushing behaviour in micro-EDM milling. In this regard, practical dielectric selection for micro-EDM applications should also consider additional machining factors, including discharge characteristics, material removal rate (MRR), tool-wear ratio (TWR), surface quality, and surface integrity. Future work should incorporate 3D modelling approaches, thermo-fluid coupling, and discharge-induced bubble dynamics to better represent realistic machining environments.

## Figures and Tables

**Table 1:** Summary of the numerical parameters used in the CFD flow simulation and particle transport analysis.

Parameter	Symbol	Value	Unit
**CFD Model**			
Flow model	–	Standard *k*–*ω*	–
Fluid viscosity	*µ*	See Table 2	Pa·s
Fluid density	*ρ*	See Table 2	kg/m^3^
Inter-electrode gap sizes	IEG	20, 25, 30	µm
Inlet velocity (liquid dielectrics)	$u_{liquids}$	0.1	cm/s
Inlet velocity (Nitrogen Gas) *	u _Nitrogen Gas_	≈83	cm/s
Tool rotational speed	*ω*	400, 600, 850	rpm
Tool direction	–	Counterclockwise	–
Boundary conditions	–	Inlet/outlet/no-slip	–
Gravity	–	Disabled	–
Flow simulation time	${\;t}_{flow}$	0:0.1:5	s
Solver type	–	Time-dependent, implicit	–
**Particle Transport Model**			
Number of injected particles	*N*_0_	200	–
Particle density	$\rho_{p}$	2710	kg/m^3^
Particle diameter	$d_{p}$	2	µm
Drag law	–	Stokes drag	–
Particle tracking time	$t_{total}$	0:0.05:5	s

(*) In the case of nitrogen gas, the inlet velocity is calculated to maintain the same mass flow rate as the dielectric water.

**Table 2:** Thermophysical properties of the dielectric fluids used in this study.

Dielectric Fluid	Dynamic Viscosity (Pa·s)	Density (kg/m^3^)
Nitrogen Gas (20 °C, 1 atm) [34]	$1.76\times 10^{-5}$	1.165
Deionized Water [30]	$1.0\times 10^{-3}$	1000
Sunflower Oil [30]	$7.9\times 10^{-3}$	920
HEDMA111 Oil	$1.87\times 10^{-3}$	781

**Table 3:** Summary of steady and quasi-steady flow metrics for the four dielectric fluids.

Fluid	Viscosity (Pa·s)	Steady Time (s)	Velocity Range (cm/s)
Deionized Water	1.0 × 10^−3^	1–1.4	1.7–3.5 × 10^−4^
HEDMA111 Oil	1.87 × 10^−3^	1.4–2.7	1.6–3.8 × 10^−4^
Nitrogen Gas	1.76 × 10^−5^	∼0.8–1.0	0.4–1.2 × 10^−2^
Sunflower Seed Oil	7.9 × 10^−3^	∼1	1.5–2.6 × 10^−4^

Note: Velocity ranges correspond to steady or quasi-steady values extracted from the late-time regime (quasi-steady interval after decay of transient peaks) across 400–850 rpm, IEG = 20–30 µm, at points A–C.

**Table 4:** Mesh analysis for the 2D CFD particle transport model at 850 rpm and 20 µm IEG using deionized water.

Mesh	Number of Elements	Average Element Quality	$t_{1/2}$ (s)	Particles Remaining at t=5 s	Escaped Particles at t=5 s (%)
Coarse mesh	4356	0.7788	1.55	39	80.5
Normal mesh	6678	0.7796	1.40	36	82.0
Fine mesh	16,778	0.7935	1.45	36	82.0

**Table 5:** Temporal sensitivity analysis for the 2D CFD particle transport model at 850 rpm and 20 µm IEG using deionized water.

Temporal Discretisation	Flow Time Step (s)	Particle Time Step (s)	$t_{1/2}$ (s)	Particles Remaining at t=5 s	Escaped Particles at t=5 s (%)
Coarse mesh	0.10	0.05	1.35	36	82.0
Normal mesh	0.05	0.025	1.50	36	82.0
Fine mesh	0.025	0.0125	1.35	36	82.0

## Data Availability

The raw data supporting the conclusions of this article will be made available by the authors on request.
